# Challenges in nasal reconstruction for facial clefts Tessier 3 bilateral and Tessier 0: a staged surgical approach case report

**DOI:** 10.1186/s40902-025-00461-0

**Published:** 2025-03-17

**Authors:** Arif Tri Prasetyo

**Affiliations:** 1 Division of Plastic Reconstructive and Aesthetic Surgery, Department of Surgery, Faculty of Medicine, Universitas Padjadjaran, Bandung, Indonesia; 2 Department of Plastic Reconstructive and Aesthetic Surgery, Universitas Padjadjaran Hospital, Sumedang, Indonesia

**Keywords:** Tessier facial cleft, Bilateral Tessier 3, Tessier 0, Nasal reconstruction, Craniofacial anomaly

## Abstract

**Background:**

Craniofacial anomalies, particularly Tessier facial clefts, present significant surgical and functional challenges. Bilateral Tessier 3 and Tessier 0 clefts are extremely rare, often requiring complex reconstructive strategies. These clefts result in severe nasal deformities, including absent nasal septum, hypertelorism, and malpositioned alae nasi, affecting both appearance and function. Due to the lack of standardized approaches in such cases, this report highlights a staged surgical reconstruction aimed at restoring nasal structure and improving facial harmony, with a 12-month follow-up showing stable nasal contour and functional airway restoration.

**Case presentation:**

A 16-year-old female presented with bilateral Tessier 3 and Tessier 0 clefts, exhibiting hypertelorism, a wide nasal dorsum, cranial displacement of the alae nasi, and an absent nasal septum. The patient underwent staged reconstruction. The first stage repositioned the alae nasi and created a functional nasal airway. In the second stage, costal cartilage was used to construct an L-shaped septal extension graft and dorsal onlay graft to restore nasal contour and stability. A subsequent procedure refined the nasal dorsum and approximated the alae nasi. Although orbital box osteotomy was planned to correct hypertelorism, the patient declined further intervention.

**Conclusion:**

This case highlights the effectiveness of a staged reconstructive approach in addressing rare craniofacial anomalies. Twelve-month postoperative follow-up confirmed the stability of nasal contour, functional airway patency, and satisfactory facial symmetry. The decision to forgo orbital box osteotomy emphasizes the role of patient-centered care in craniofacial surgery. This case provides valuable insights for optimizing reconstructive techniques in complex facial clefts.

**Supplementary Information:**

The online version contains supplementary material available at 10.1186/s40902-025-00461-0.

## Introduction

Craniofacial abnormalities are primarily identified through their physical characteristics. These malformations are typically classified based on clinical or anatomical criteria, often without considering their developmental stage or underlying pathology. With an estimated incidence of 1 in 10,000,000 births, they may occur sporadically or as part of a genetic anomaly sequence. Embryologically, these malformations arise from the incomplete fusion of the medial nasal prominences [1–3].

Facial clefts are unique anomalies, with each case presenting distinct characteristics. The Tessier classification system, introduced by Paul Tessier in 1976, categorizes facial clefts based on the anatomical location of defects in both soft tissue and bone. This system assigns numbers (0–14) to each craniofacial cleft based on its position relative to the sagittal midline and the orbit, noting a relationship between the soft tissues and underlying bone [4]. Due to the variability in clinical presentations, even among patients with the same classification, each facial cleft case can offer unique insights and serve as an interesting case report [5].

Tessier 0 facial cleft, also known as the median cleft, is a facial cleft with various characteristics. A true median cleft is often marked by the absence of the nasal septum. Other features that may be present include excess tissue in the nasal dorsum due to the missing septum and an increased distance between the alae nasi. Hypertelorism can also be a distinguishing characteristic. Additionally, the lip may sometimes appear retracted due to the median cleft anomaly [6].

Tessier 3 bilateral facial cleft is a rare craniofacial anomaly characterized by a cleft extending from the upper lip to the lower eyelid, affecting the nasomaxillary and zygomatic regions. The condition often results in a widened nasal base, significant midfacial flattening, and an increased distance between the inner and outer corners of the eyes, leading to telecanthus. Associated anomalies may include incomplete formation of the lower eyelid, coloboma, or defects in the lacrimal system. The involvement of both soft tissue and underlying skeletal structures presents substantial functional and aesthetic challenges, necessitating complex reconstructive approaches to restore facial symmetry and function [7].

This case report will discuss the nasal reconstruction approach in patients with bilateral Tessier 3 and Tessier 0 facial clefts. Due to the rarity of this specific combination of craniofacial anomalies, there is limited literature on standardized surgical techniques for managing such cases. The complexity of these clefts presents significant challenges in both functional and aesthetic reconstruction, requiring a tailored approach to restore nasal structure and facial symmetry. Therefore, this case report aims to provide valuable insights and serve as a reference for further advancements in surgical techniques for facial cleft repair.

## Presentation of case

**Fig. 1:**
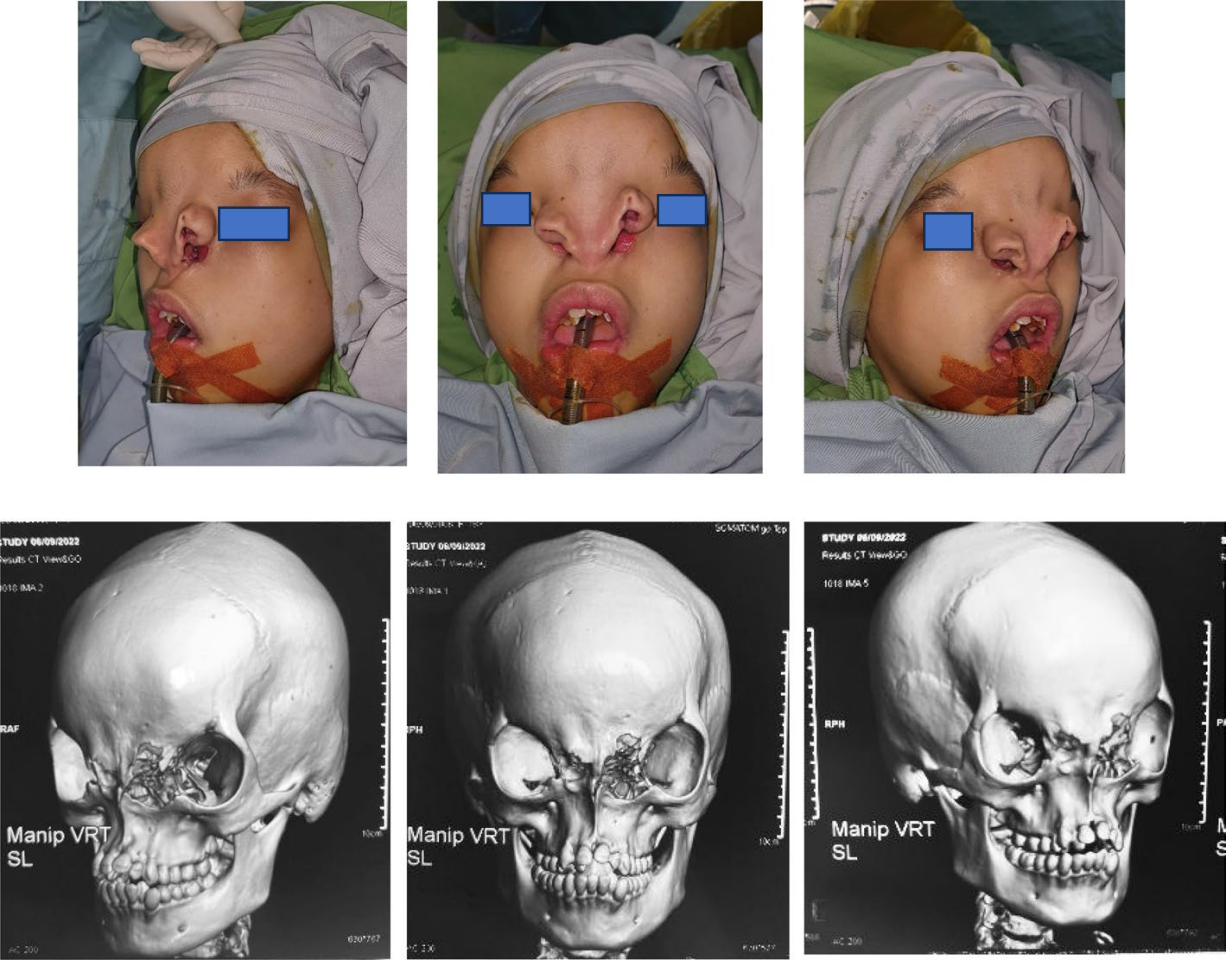
Clinical and radiological manifestations of bilateral Tessier 3 and Tessier 0 facial clefts. **A** Preoperative frontal view showing bilateral Tessier 3 and Tessier 0 clefts with significant cranial displacement of the alae nasi, widened nasal dorsum, and hypertelorism. **B** Lateral preoperative view highlighting the absence of nasal septum and flat midfacial profile. **C** 3D computed tomography (CT) reconstruction illustrating the complete absence of the nasal septum, widened interorbital distance measuring 38 mm, and asymmetry in the maxillary region. These findings provided critical information for surgical planning and highlighted the complexity of the reconstruction

A 16-year-old female patient presented with a congenital facial anomaly characterized by bilateral Tessier 3 and Tessier 0 clefts (Fig. 1). Clinically, the patient exhibited significant nasal deformity with the absence of the nasal septum, widened nasal dorsum, and increased distance between the alae nasi, consistent with the features of a median cleft. Additionally, the patient displayed hypertelorism and midfacial flattening, with a wide intercanthal distance. The maxillary region showed asymmetry with malocclusion and dental crowding, along with a defect extending to the lower eyelids and orbital rim. Despite the severity of the anomaly, the patient had no significant history of systemic illness or prior surgical intervention. A comprehensive evaluation, including 3D CT imaging, confirmed the extent of the craniofacial defects, highlighting the challenges in reconstructive planning. Comprehensive 3D computed tomography (CT) imaging revealed significant craniofacial anomalies, including complete absence of the nasal septum, widened interorbital distance measuring 38 mm (consistent with hypertelorism), and cranial displacement of the alae nasi. The maxillary region displayed asymmetry with malocclusion and crowding of teeth. These imaging findings provided critical insights for surgical planning, confirming the extent of bony and soft tissue involvement and the need for a staged reconstructive approach. This case demonstrates the unique combination of rare facial clefts, requiring a multidisciplinary approach for functional and aesthetic restoration.

The bilateral Tessier 3 cleft in this patient presented with several unique and complex anatomical features, setting it apart from typical cases. One of the most notable characteristics was the malposition of the alae nasi, which were displaced cranially, far from their normal anatomical location. This cranial displacement significantly altered the nasal appearance and structure, creating both functional and aesthetic challenges. Additionally, the patient exhibited an unusual configuration of the nasal openings. There were two visible nasal passages: an upper opening that was blind-ended and non-functional, and a lower opening that was the sole pathway used by the patient for breathing. This abnormal configuration not only affected nasal airflow but also posed challenges for surgical correction and functional restoration.

This study was conducted in accordance with the ethical standards of the institution, and IRB approval was not required as it is a single case report. Written informed consent for publication of their clinical details and/or clinical images was obtained from the patient. A copy of the consent form is available for review by the Editor of this journal.

The surgical reconstruction was performed in three stages, with a 6-month interval between the first and second stages to ensure proper soft tissue healing and stabilization. The first stage prioritized establishing a functional nasal airway and repositioning the alae nasi to improve nasal alignment. The second stage focused on reconstructing the absent nasal septum and restoring nasal contour using costal cartilage grafts. A final stage involved dorsal refinement and medial repositioning of the alae nasi to optimize nasal symmetry. This sequential approach minimized surgical risks such as cartilage warping and tissue necrosis, allowing for gradual adaptation of nasal structures.

The first surgical intervention was meticulously planned to address these unique deformities. The primary focus of the procedure was the repositioning of the alae nasi to their correct anatomical location, moving them downward to achieve a more natural alignment with the midface. This repositioning aimed to improve both the functional and aesthetic aspects of the nose. In addition, the blind-ended upper nasal opening was surgically perforated and connected to the functional lower nasal passage. This step was critical in creating a unified and functional nasal airway, allowing the patient to breathe through the nose in a manner comparable to that of a normal individual.

**Fig. 2:**
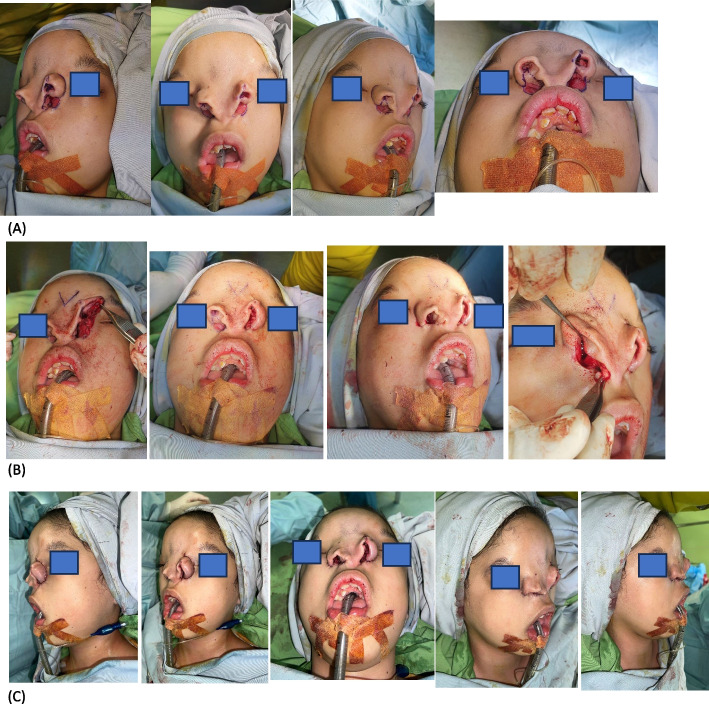
First-stage surgical reconstruction: alae nasi repositioning and airway creation. **A** Surgical design and markings outlining the repositioning of the alae nasi and creation of a functional nasal airway. **B** Intraoperative image demonstrating cranial displacement of the alae nasi and the blind-ended superior nasal opening. Partial excision was performed to create an opening that could be connected to the functional lower nasal passage. **C** Immediate postoperative result showing successful repositioning of the alae nasi to their anatomical location and the establishment of a unified functional nasal airway, allowing effective nasal breathing

The overarching goal of the surgery was twofold: first, to achieve a proper anatomical position for the alae nasi, and second, to establish functional nasal passages that would enable the patient to breathe through the nose comfortably and effectively. This surgical approach not only addressed the immediate functional issues but also laid the groundwork for future interventions aimed at further improving the patient's quality of life and nasal aesthetics. The combination of these unique anatomical features and the complexity of the surgical correction highlights the challenges and innovations required in managing rare craniofacial anomalies like bilateral Tessier 3 clefts (Fig. 2).

**Fig. 3:**
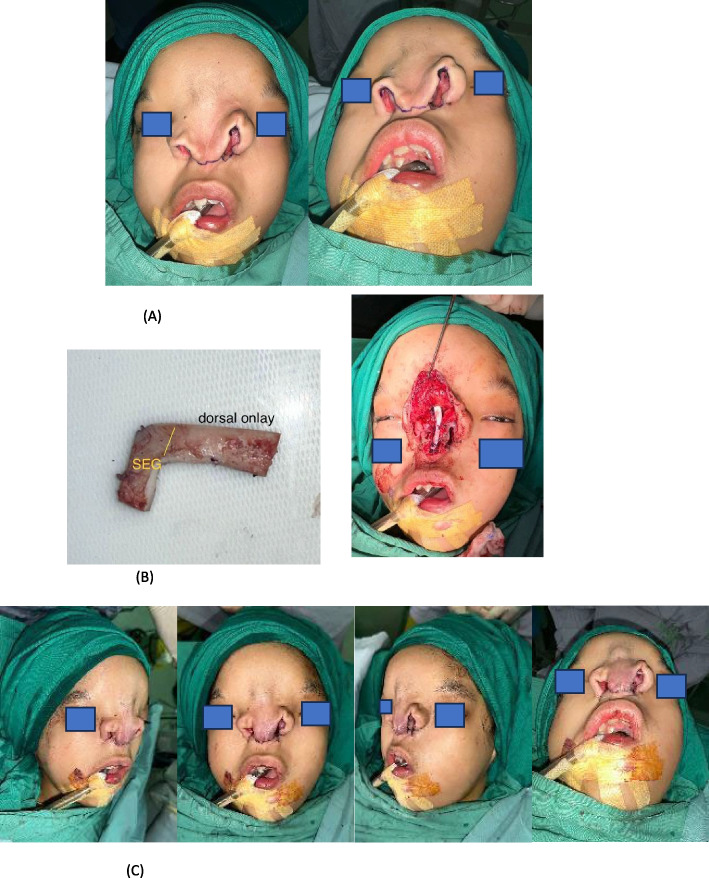
Second-stage reconstruction: costal cartilage grafting for septal and dorsal support. **A** Harvesting of costal cartilage from the right sixth rib. The cartilage was sculpted into an L-shaped graft comprising a septal extension graft and dorsal onlay graft. **B** Intraoperative placement of the L-shaped graft. The septal extension graft provided central support, while the dorsal onlay graft restored nasal height and contour. **C** Immediate postoperative appearance demonstrating improved nasal contour and projection. The reconstructed nasal septum provided enhanced structural stability, though minor asymmetry of the nasal dorsum was noted during follow-up

In the subsequent stage of surgery, the primary focus was on addressing the Tessier 0 anomaly, specifically reconstructing the absent nasal septum. This was achieved by creating an L-shaped septal extension graft and a dorsal onlay graft, with the distal end of the graft meticulously positioned on the nasal bone to provide structural support and improve nasal contour. The procedure was executed as planned, and the surgical team was confident in achieving the desired structural improvements. However, during the closure process and subsequent follow-ups in the outpatient clinic, it was observed that the nasal shape was not yet optimal (Fig. 3).

**Fig. 4:**
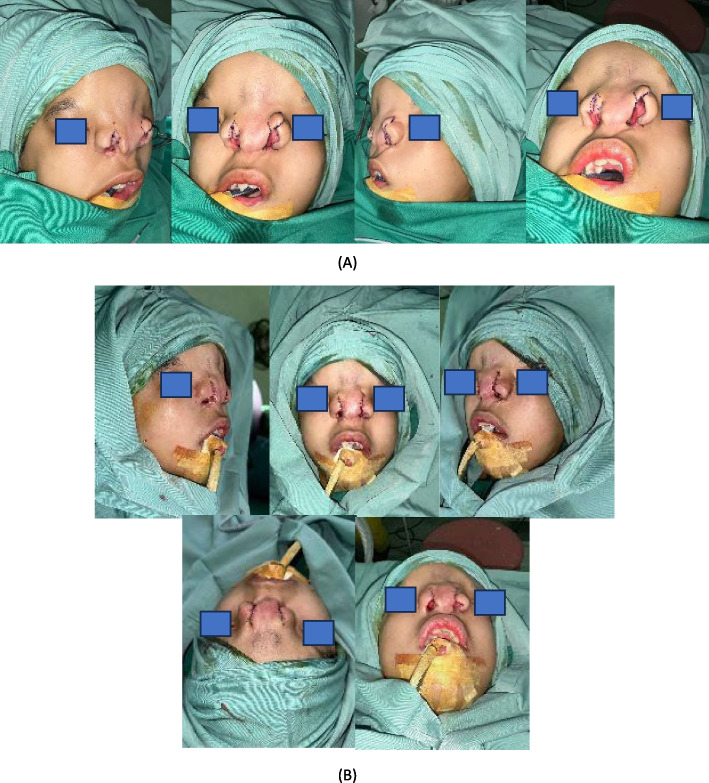
Third-stage refinement: medial repositioning of alae nasi and dorsal reduction. **A** Surgical design for medial repositioning of the alae nasi to reduce the interalar distance. Wedge excisions were planned at the alar base, and excess soft tissue was marked for removal from the dorsal nasal area to improve nasal contour. **B** Intraoperative image showing rotational flaps on both alae nasi for precise repositioning and dorsal tissue reduction. **C** Immediate postoperative outcome highlighting enhanced nasal symmetry, reduced interalar distance, and improved dorsal contour. The surgical scar was strategically placed along the dorsum nasi to minimize visibility

The primary concern noted was the imbalance in the dorsum nasi, which was caused by the significant distance between the right and left alae nasi. This disproportion created an asymmetry in the nasal structure, detracting from the aesthetic results of the reconstruction. To address this, an additional surgical intervention was deemed necessary. This involved the removal of excess soft tissue from the nasal dorsum, which had contributed to the asymmetry, and the approximation of the right and left alae nasi to reduce the interalar distance and create a more harmonious nasal shape. This stage also includes ala nasi repositioning on the medial side (Fig. 4).

The secondary procedure was carefully staged to ensure the structural integrity of the reconstructed nose was preserved, avoiding overcorrection or compromising the functional outcomes. The scar from this procedure was strategically placed along the dorsum nasi, serving as a clear and defined boundary for the nasal contour while minimizing its visibility. This meticulous approach aimed to achieve both functional and aesthetic improvements, providing the patient with a more natural nasal appearance while ensuring that the structural foundation of the nose remained uncompromised. This staged reconstruction highlights the complexity of managing craniofacial anomalies and the importance of iterative surgical planning to address evolving anatomical and aesthetic considerations effectively (Fig. 4).

**Fig. 5:**
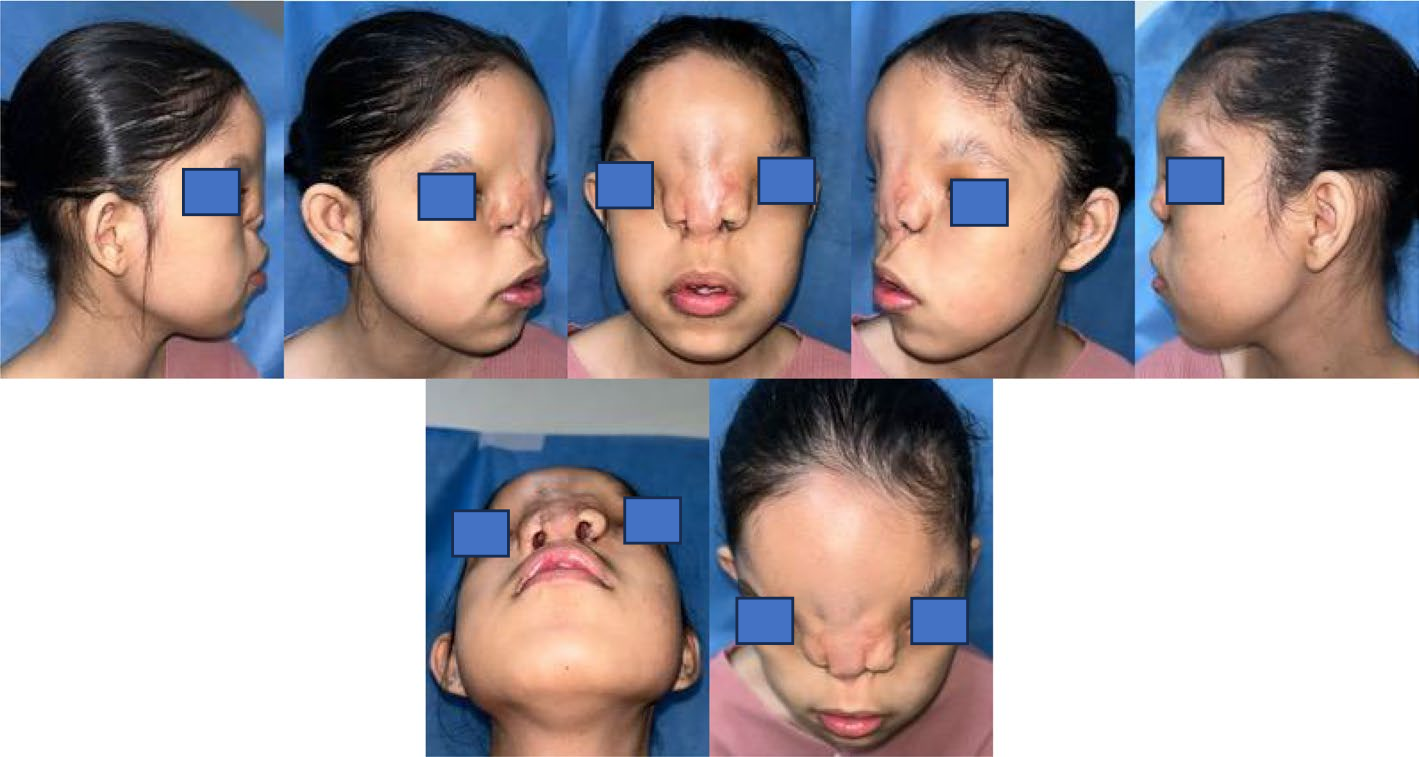
Final postoperative appearance at 12-month follow-up. **A** Frontal view showing improved nasal symmetry, well-defined nasal dorsum, and symmetrical alae nasi positioning. The interalar distance was significantly reduced compared to the preoperative state, contributing to balanced facial proportions. **B** Lateral view illustrating adequate nasal projection and stable contour. **C **Basal view revealing functional and symmetrical nasal openings with a patent airway. Minor dorsal irregularities remain, primarily due to scarring from previous interventions. The patient declined further refinement procedures, including orbital box osteotomy, citing satisfaction with the current aesthetic and functional outcomes

The current nasal appearance of the patient demonstrates significant improvement following the staged reconstructive surgeries (Fig. 5). The nasal dorsum is now relatively straight and well-defined, though minor irregularities in contour remain visible, likely due to the scarring from prior interventions. The nasal tip appears broad with limited projection, contributing to a slightly flattened appearance. The alae nasi have been repositioned symmetrically and are better aligned with the midface; however, the interalar distance remains relatively wide, which impacts the overall nasal proportions. The nasal openings are now functional and centrally positioned, enabling the patient to breathe effectively, a significant improvement from the preoperative condition. Despite these advancements, some aesthetic refinements could still be considered, particularly in improving nasal tip projection and harmonizing the overall proportions.

To achieve more balanced facial proportions and create a more favorable foundation for nasal reconstruction, particularly in the dorsum nasal area, a comprehensive surgical strategy was proposed. This plan involved addressing the patient’s telecanthus and hypertelorism, which are significant contributors to the overall facial asymmetry and disproportion. The recommended procedure was an orbital box osteotomy, a complex yet effective surgical technique designed to correct the widened interorbital distance and bring the orbits into a more anatomically harmonious alignment. This correction would not only improve the aesthetic symmetry of the face but also provide a more stable and proportional base for subsequent nasal reconstruction.

Despite the potential benefits of the orbital box osteotomy in enhancing both functional and aesthetic outcomes, the patient expressed concerns regarding the high risks associated with the procedure. Due to its invasive nature and potential complications, the patient ultimately declined this intervention. This decision highlights the delicate balance between achieving optimal surgical outcomes and respecting the patient’s preferences and tolerance for risk. The surgical team remains committed to exploring alternative approaches that align with the patient’s goals while maintaining safety and minimizing potential risks. This case underscores the importance of individualized treatment planning in complex craniofacial reconstruction.

## Discussion

This patient was diagnosed with bilateral Tessier 3 facial cleft and Tessier 0. The diagnosis of Tessier 3 was based on the clinical features observed in the patient, including cranial displacement of the alae nasi, significant widening of the nasal base, hypertelorism, and midfacial flattening. These clinical findings are consistent with the classification of facial clefts introduced by Paul Tessier, which categorizes craniofacial anomalies based on their anatomical location and extent. In patients with a Tessier 3 facial cleft, typical abnormalities include a disruption in the nasomaxillary and zygomatic regions, leading to deformities in both soft tissue and underlying skeletal structures [4–7].

The diagnosis of Tessier 0 in this patient was based on clinical examination, which revealed key features such as the absence of the nasal septum and hypertelorism. Tessier 0, often referred to as the median cleft, is characterized by anomalies occurring along the midline of the face. This rare craniofacial anomaly is not limited to soft tissue abnormalities but can also involve underlying skeletal structures and even adjacent organs. In this case, the absence of the nasal septum resulted in significant anatomical disruption, contributing to a widened nasal dorsum and altered nasal functionality. Additionally, the hypertelorism, marked by an increased distance between the orbits, further supported the diagnosis. These features are among the hallmark clinical signs of Tessier 0, providing a clear basis for its classification and highlighting the complexity of reconstructing both the functional and aesthetic aspects of the face in such cases [8–10].

 The primary concern of the patient was the deformity of the nose, which became the focus of the reconstructive interventions. In the first stage, the surgical team repositioned the alae nasi to their proper anatomical location and created an opening in the nasal structure to establish functional nasal airflow. This initial procedure successfully relocated the lateral alae nasi, laying the foundation for subsequent reconstructive efforts. The next stage involved addressing the absence of the nasal septum and reconstructing the dorsum using costal cartilage. The costal cartilage was harvested and shaped into an L-shaped graft, serving as both a septal extension graft and a dorsal onlay. This cartilage graft provided the necessary structural support and contour for the collapsed nasal framework caused by the loss of the nasal septum.

This staged approach not only restored nasal functionality but also aimed to achieve an aesthetically pleasing nasal profile. The use of costal cartilage is widely recognized in the literature as a robust and reliable material for nasal reconstruction, especially in cases of severe structural deficiency. According to Grosu-Bularda et al. (2016), costal cartilage grafts are ideal for restoring nasal framework stability and achieving durable results in complex cases. Similarly, Pradhan (2024) emphasizes the role of L-shaped grafts in maintaining both aesthetic and functional outcomes in patients with severe nasal deformities. These techniques highlight the importance of individualized, staged reconstruction in addressing both the structural and cosmetic needs of patients with craniofacial anomalies, as seen in this case [11, 12].

In the next stage of reconstruction, medial repositioning of the alae nasi was performed to achieve better nasal harmony. The technique utilized involved a rotational flap on the right and left alae nasi, allowing for improved alignment and symmetry. Additionally, a wedge excision at the alar base was conducted to reduce the width of the nasal base and bring the alae nasi closer together. This approach aimed to minimize the excessive interalar distance, thereby enhancing the overall nasal proportions and aesthetics. Furthermore, tissue reduction in the central nasal area along the dorsal nasal margin was performed to eliminate excess tissue that could contribute to asymmetry or imbalance in the nasal contour. By refining the nasal dorsum and optimizing the positioning of the alae nasi, this procedure sought to create a more natural and harmonious nasal structure [9, 13].

For the next stage of treatment, the patient was planned to undergo orbital box osteotomy to correct hypertelorism. The procedure involved cutting and repositioning the orbital boxes medially by reducing the frontal bone, aiming to create better facial harmony and improve the proportions of the nose and eyes [14, 15]. However, the patient declined the procedure, feeling satisfied with the current nasal shape.

The patient expressed high levels of satisfaction regarding both aesthetic and functional outcomes. Using a validated patient-reported outcome measure (PROM) specific to facial reconstructive surgery, the patient rated overall satisfaction with a nasal appearance at 8 out of 10, citing significant improvement in nasal symmetry and contour. Functionally, the patient reported normal nasal breathing without obstruction, contributing positively to daily activities and overall well-being.

Regarding quality of life, assessments using the Facial Clinimetric Evaluation (FaCE) scale indicated improvements in self-esteem and social interactions, reflecting enhanced psychosocial outcomes post-reconstruction. The patient resumed regular schooling and reported increased confidence in public settings, highlighting the broader impact of surgical success beyond physical restoration.

Long-term evaluation at 12 months demonstrated stable nasal contour with no signs of graft resorption or airway compromise. Although minor dorsal contour irregularities persisted, the patient declined further refinements, expressing contentment with the current outcome. This case underscores the significance of incorporating patient-centered metrics in evaluating reconstructive success, reinforcing the value of staged approaches in addressing complex craniofacial anomalies.

Despite successful functional and aesthetic outcomes, several limitations should be acknowledged. The patient’s refusal to undergo orbital box osteotomy limited the correction of hypertelorism, which may have further enhanced overall facial harmony. Additionally, the follow-up period of 12 months may not fully capture the long-term stability of the costal cartilage grafts or potential late-onset complications. Future research should focus on evaluating long-term outcomes of staged nasal reconstruction in similar cases, with emphasis on the durability of costal cartilage grafts and potential non-invasive alternatives for addressing hypertelorism. Multicenter studies involving larger patient cohorts could also contribute to establishing standardized protocols for managing rare craniofacial clefts.

## Conclusion

This case highlights the complexity of managing rare craniofacial anomalies, particularly the combination of bilateral Tessier 3 and Tessier 0 facial clefts. The patient presented with significant nasal deformities, including the absence of the nasal septum, hypertelorism, and mispositioned alae nasi, which required a staged surgical approach for both functional and aesthetic restoration. Initial interventions focused on repositioning the alae nasi and creating a functional nasal airway, followed by structural reconstruction using costal cartilage grafts to provide nasal support. Additional procedures aimed to refine the nasal dorsum and achieve better facial symmetry by reducing excessive tissue and approximating the alae nasi.

Despite the proposed orbital box osteotomy to further correct hypertelorism and improve facial proportions, the patient opted against it, highlighting the importance of individualized treatment planning that balances surgical benefits with patient preferences. The staged approach proved effective in restoring nasal function and achieving a more natural facial contour, demonstrating the necessity of multidisciplinary collaboration in craniofacial reconstruction. This case serves as a valuable reference for optimizing reconstructive strategies in similar complex facial cleft cases.

## Supplementary Information

Supplementary Material 1.

## Data Availability

No datasets were generated or analysed during the current study.
