# Supplementary material for: Challenges in nasal reconstruction for facial clefts Tessier 3 bilateral and Tessier 0: a staged surgical approach case report
Source: Maxillofac Plast Reconstr Surg. 2025 Mar 17;47(1):7. doi: 10.1186/s40902-025-00461-0 (PMC11914444; doi:10.1186/s40902-025-00461-0)
Supplement: Supplementary file 1 — Supplementary Material 1. [file 40902_2025_461_MOESM1_ESM.docx]

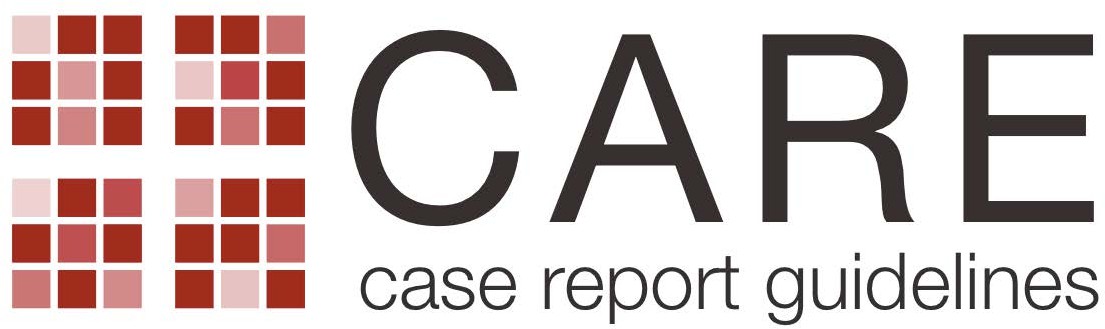
CARE Checklist of information to include when writing a case report
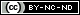


**Topic Item Checklist item description Reported on Line**

**Title 1** The diagnosis or intervention of primary focus followed by the words “case report” . . . . . . . . . . . . . . . . . . 1

***Key Words 2*** *2 to 5 key words that identify diagnoses or interventions in this case report, including "case report"* 2

***Abstract***

***(no references)***

***3a*** *Introduction: What is unique about this case and what does it add to the scientific literature? Background 5-11*

***3b*** *Main symptoms and/or important clinical findings . . . . . . . . . . . . . . . . . . . . . . . . . . . . . . . . . . . . . . . . . . . . . . . . . . .*  13 - 21

***3c*** *The main diagnoses, therapeutic interventions, and outcomes* 13 - 21

***3d*** *Conclusion—What is the main “take-away” lesson(s) from this case?* 23 - 28

***Introduction 4*** *One or two paragraphs summarizing why this case is unique (****may include*** *reference****s****) 61-67*

***Patient Information 5a*** *De-identified patient specific information 71-282*

***5b*** *Primary concerns and symptoms of the patient 71-282*

***5c*** *Medical, family, and psycho-social history including relevant genetic information*  *71-282*

**5d** Relevant past interventions with outcomes *71-282*

**Clinical Findings**

**Timeline**

**Diagnostic Assessment**

**Therapeutic Intervention**

**Follow-up and Outcomes**

1. Describe significant physical examination (PE) and important clinical findings *71-282*
2. Historical and current information from this episode of care organized as a timeline *71-282*

**8a** Diagnostic testing (such as PE, laboratory testing, imaging, surveys). *71-282*

**8b** Diagnostic challenges (such as access to testing, financial, or cultural) *71-282*

**8c** Diagnosis (including other diagnoses considered) *71-282*

**8d** Prognosis (such as staging in oncology) where applicable *71-282*

**9a** Types of therapeutic intervention (such as pharmacologic, surgical, preventive, self-care) . . . . . . . . . . . . . . . . . *71-282*

**9b** Administration of therapeutic intervention (such as dosage, strength, duration) *71-282*

**9c** Changes in therapeutic intervention (with rationale) *71-282*

**10a** Clinician and patient-assessed outcomes (if available) *71-282*

**10b** Important follow-up diagnostic and other test results *71-282*

**10c** Intervention adherence and tolerability (How was this assessed?) *71-282*

**10d** Adverse and unanticipated events N/A

**Discussion 11a** A scientific discussion of the strengths AND limitations associated with this case report 285-336

**11b** Discussion of the relevant medical literature **with references** 285-336

**11c** The scientific rationale for any conclusions (including assessment of possible causes) 285-336

**11d** The primary “take-away” lessons of this case report (without references) in a one paragraph conclusion 338 - 354

**Patient Perspective 12** The patient should share their perspective in one to two paragraphs on the treatment(s) they received . . . . 338 - 354

**Informed Consent 13** Did the patient give informed consent? Please provide if requested . . . . . . . . . . . . . . . . . . . . . . . . . . . . . . . . . . . . . . **Yes** √ **No
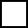
**
